# Tip-enhanced photoluminescence of monolayer MoS_2_ increased and spectrally shifted by injection of electrons

**DOI:** 10.1515/nanoph-2023-0025

**Published:** 2023-04-06

**Authors:** Jizhou Wang, Zehua Han, Zhe He, Kai Wang, Xiaohui Liu, Alexei V. Sokolov

**Affiliations:** Institute for Quantum Science and Engineering, Texas A&M University, College Station, TX 77843, USA; Department of Medical Engineering, California Institute of Technology, Pasadena, CA 91125, USA; Department of Physics, University of Texas at Austin, Austin, TX 78712, USA; Baylor Research Innovative Center, Baylor University, Waco, TX 76798, USA

**Keywords:** defect, monolayer MoS_2_, plasmon-induced electron injection, tip-enhanced photoluminescence

## Abstract

Using tip-enhanced photoluminescence (TEPL), we investigate micron-size monolayer MoS_2_ flakes. In a sequence of studies, we apply various voltages between the Ag-coated nano-tip and the MoS_2_ flakes and observe an intriguing result. During the TEPL measurement, we observe that the photoluminescence spectrum is blue shifted and the overall signal intensity is increased. We attribute this behavior to plasmon-induced electron injection into MoS_2_. Additionally, when the tip is negatively biased with respect to the sample during the TEPL measurement, the nonuniform TEPL images of MoS_2_ monolayer flakes containing defects are gradually changed to be uniform that reach saturation. We verify that this saturation state in TEPL can last over half a year.

## 1 Introduction

Photoluminescence (PL) of monolayer transition metal dichalcogenides (TMDCs) reveals peculiar nanostructures such as heterojunctions [1, 2], boundaries [3], and defects [3–9]. Because the PL spectrum of TMDCs with defects differs slightly from that of TMDCs without defects in both the intensity and peak position, the PL imaging of TMDCs often appears nonuniform [8–10]. With electric fields and hot electron injections [11], it is possible to manipulate the optical response of imperfect crystal structures to approximate that of TMDCs, which suggests that the nonuniform optical response caused by defects may be “fixed.” Because of their small size, however, defects are likely to affect the PL on the length scale that is much smaller than the optical diffraction limit, making their precise detection and manipulation difficult.

Tip-enhanced photoluminescence (TEPL) [12] solves both problems by enhancing PL emission and resolving subdiffraction structures through localized surface plasmon resonances (LSPR) of a metal nano-tip. The enhancement factor of TEPL depends on the size, shape, and material of the tip and was reported to be as high as 10^4^ [13]. The spatial resolution of TEPL is approximately equal to the apex size of the tip, which is ∼20 nm in our experiment. We note that in certain cases, the spatial resolution of a system employing a metallized tip can be at least an order of magnitude smaller than the apex size [14, 15]. However, this is not expected to be the case in the present experiment.

Among TMDCs, molybdenum disulfide (MoS_2_) is a popular subject in research due to its remarkable electronic and optical properties [16–18]. Monolayer MoS_2_ is a direct-gap semiconductor with stronger PL compared to indirect-gap materials [18–20]. In this paper, we measure the TEPL of monolayer MoS_2_ flakes using a resonant laser wavelength of 532 nm. We confirm that TEPL is capable of identifying defects and boundary areas with subwavelength spatial resolution [21] and show that the TEPL signals of the defects and the boundary are weaker and redshifted in comparison to defect-free areas of monolayer MoS_2_. Further, we experimentally demonstrated that these defects that cause weak optical response can be fixed by TEPL scanning.

The localized surface plasmon of the tip induces hot electron injection when it comes into contact with MoS_2_. This phenomenon has been demonstrated by the observation of a transition from excitons to negative trions in monolayer WS_2_ [22]. Plasmon-induced hot-electron injection influences the optical response of defects. We hypothesize that upon filling up the defect area, hot electrons occupy the conduction band of the defect, resulting in a blue shift of the PL. As the blue-shift effect compensates the red-shift effect caused by the defect, the imperfect optical response of MoS_2_ becomes uniform. Owing to the small tip size and fast hot electron injection, this method allows fast tuning of the PL of local defects. We study the blue-shift effect of defects in MoS_2_ and demonstrate that the changes in PL last over 6 months. Additionally, we prove that applying bias voltages can further enhance hot-electron injection. In our work, TEPL serves as a versatile technique for detecting and manipulating the small defects in TMDCs and fixing the nonuniform optical response caused by defects.

## 2 Methods and results

**Figure 1:**
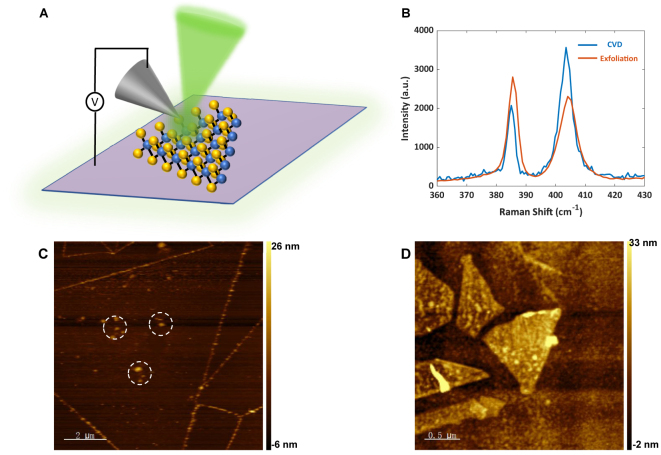
Schematic of the experiment setup and characterization of monolayer MoS_2_ samples by Raman spectroscopy and AFM. (A) Schematic of tip-enhanced photoluminescence (TEPL). MoS_2_ samples are placed on an SiO_2_/Si substrate. Au/Ag-coated tips were used for TEPL scanning. Voltages are added between the tip and the substrate. An objective (100×, NA 0.7) was used to focus the 532 nm laser on the tip and collects TEPL signals. (B) Raman spectra of CVD-grown (blue) and mechanically exfoliated (red) monolayer MoS_2_. The two Raman peaks of the CVD-grown sample locate at 385.51 cm^−1^ ( $E_{2g}^{1}$ ) and 404.10 cm^−1^ (*A*_1*g*_), and 385.03 cm^−1^ ( $E_{2g}^{1}$ ) and 403.52 cm^−1^ (*A*_1*g*_) of mechanically exfoliated flakes. (C) AFM image of a CVD-grown MoS_2_ monolayer on a 300 nm SiO_2_/Si substrate. The white dashed circles represent contaminated areas, such as residual sulfur. (D) AFM image of mechanically exfoliated MoS_2_ monolayer flakes on a 285 nm SiO_2_/Si substrate. There are five flakes shown in the scanned area.

TEPL experiments were conducted using an HORIBA-AIST-NT system. This setup is capable of performing standard AFM imaging that measures the surface structures about a MoS_2_ flake with nanoscale resolution, while simultaneously measuring its PL spectrum [23]. The scheme of the TEPL setup is illustrated in Figure 1A. The 532 nm laser beam (600 μW) is focused on the Ag-coated tip by a 100× objective (NA = 0.70). The MoS_2_ flakes are deposited on a SiO_2_/Si substrate. The number of the MoS_2_ layers are determined by their Raman spectra, which are shown in Figure 1B. The distances between two Raman characteristic peaks of CVD-grown (blue) and mechanically exfoliated (red) MoS_2_ flakes are measured to be 18.59 cm^−1^ and 18.49 cm^−1^, respectively. They indicate that these MoS_2_ flakes are monolayer according to previous reports [24–27]. The Au or Ag tip contacts the MoS_2_ flake and scans across the flake surface with a step size of 100 nm (CVD) and 50 nm (mechanically exfoliated). At each step, we collect the PL signal with the same objective and measure the spectrum with HORIBA’s Synapse EMCCD detector.

**Figure 2:**
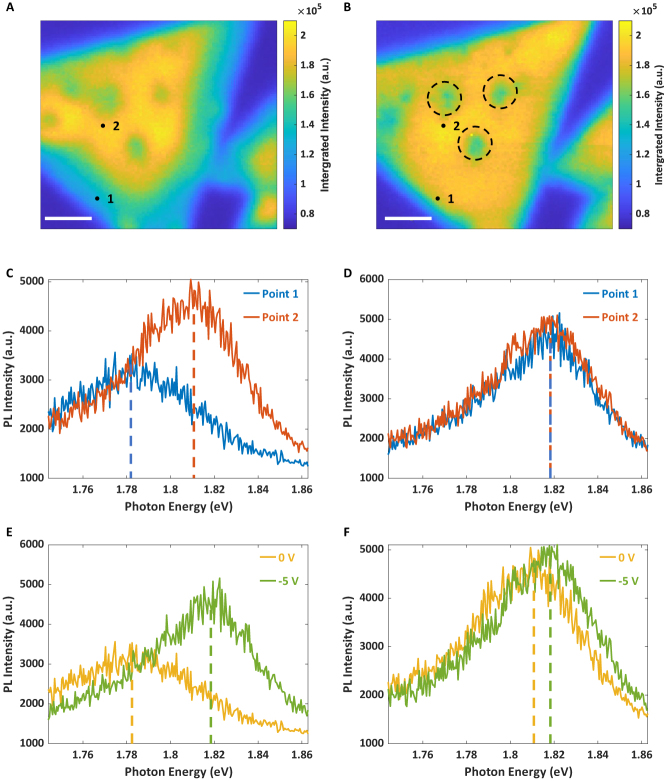
TEPL images and spectra of a CVD-grown monolayer MoS_2_ flake under different bias voltages. (A, B) TEPL images of the CVD-grown monolayer MoS_2_ flake (Figure 1C) under bias voltages of 0 V and −5 V, respectively. The step size of scan is 100 nm. The colors of pixels represent the integrated intensity of TEPL over 0.5 s. The two black dots represent position 1 and 2. Black circles represent contaminated areas consistent to Figure 1C. The scalebar is 2 μm. (C, D) TEPL spectra collected at position 1 and 2 labelled in (A, B) respectively. Each spectrum is averaged over adjacent four spatial pixels (2 × 2). The dashed lines indicate the peak centers of each spectrum. Peak centers labelled by the blue and red are 1.782 eV and 1.814 eV in (C) (0 V); 1.818 eV and 1.818 eV in (D) (−5 V). (E, F) are TEPL spectra at position 1 (E) and position 2 (F) under two different voltages.

In the first part of this research, we examined TEPL of a CVD-grown monolayer MoS_2_. According to the Raman (Figure 1B) and AFM results (Figure 1C), the flake thickness is mostly uniform, but a few areas at the edge and center are thicker, possibly due to residual sulfur during the CVD growth. The TEPL measurements were conducted by scanning the Au tip across the MoS_2_ surface in contact mode for 0.5 s per step. We performed TEPL imaging while applying different bias voltages to the tip to illustrate the effect of bias voltages to the PL spectrum of the monolayer MoS_2_. An electrical bias was applied to the tip, and the SiO_2_/Si substrate was grounded during the scanning process. To serve as a reference, 0 V bias voltage was applied in the first scan (Figure 2A). The TEPL imaging display nonuniform distribution of both the intensity and the peak center with an MoS_2_ monolayer flake. As shown in Figure 2C, the TEPL intensity near the edges is weaker than at the center, and the corresponding PL spectrum exhibits a red shift in comparison to that of MoS_2_ (1.814 eV). Nevertheless, after several measurements under nonzero bias voltages, the TEPL spectra became almost identical over the flake. A TEPL image was obtained under a bias voltage of −5 V, as shown in Figure 2B. The lumpy areas near the flake center (black dashed circles shown in Figure 2B) correspond to the protruded areas labelled in Figure 1C (white dashed circles). Figure 2D shows the spectra acquired at positions 1 and 2 under the −5 V bias voltage. Comparisons of the PL spectra of positions 1 and 2 with 0 and −5 V bias voltages are shown in Figure 2E and F, respectively. Under the 0 V scan, the PL signal at position 1 was weaker and red shifted according to that at position 2. In the course of the scan with the −5 V bias voltage, the PL signal at position 1 became stronger and blue shifted to resemble the spectrum at position 2. In contrast, the TEPL spectrum at position 2 shows few changes. Upon saturation, which means that the TEPL spectrum becomes the same throughout the MoS_2_ monolayer flake, the PL will no longer vary under bias voltages.

**Figure 3:**
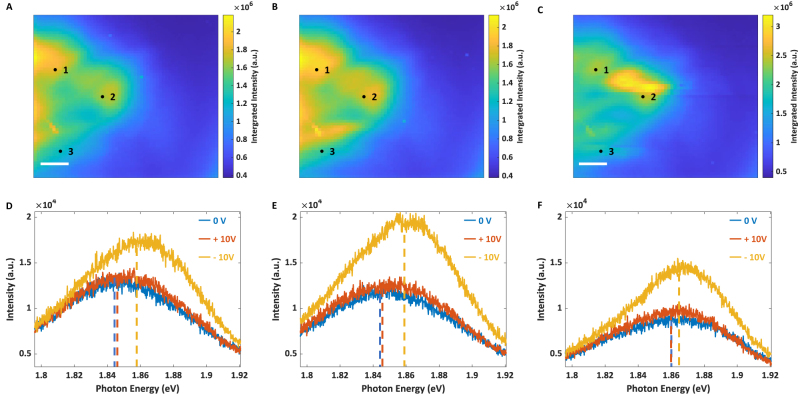
TEPL images and spectra of mechanically exfoliated monolayer MoS_2_ samples under diferent bias voltages. (A–C) TEPL images of monolayer MoS_2_ flakes prepared via mechanically exfoliation. The acquisition time is set as 2 s. The tip scans with 50 nm step-size under bias voltages of 0 V, +10 V, and −10 V, respectively. The voltages are applied to the substrate, and the Ag-coated tip was connected to the ground. The scalebar is 0.5 μm. (D–F) TEPL spectra collected under different voltages at position 1, 2, and 3, as are shown in (A), (B), and (C) respectively. Each spectrum is averaged over adjacent four spatial pixels (2 × 2). The dashed lines indicate the centers of the peaks of each spectrum. Peak centers labelled by the blue, red, and yellow lines are 1.845 eV, 1.847 eV, and 1.854 eV in (D) 1.845 eV, 1.846 eV, and 1.859 eV in (E) and 1.860 eV, 1.860 eV, and 1.863 eV in (F).

**Figure 4:**
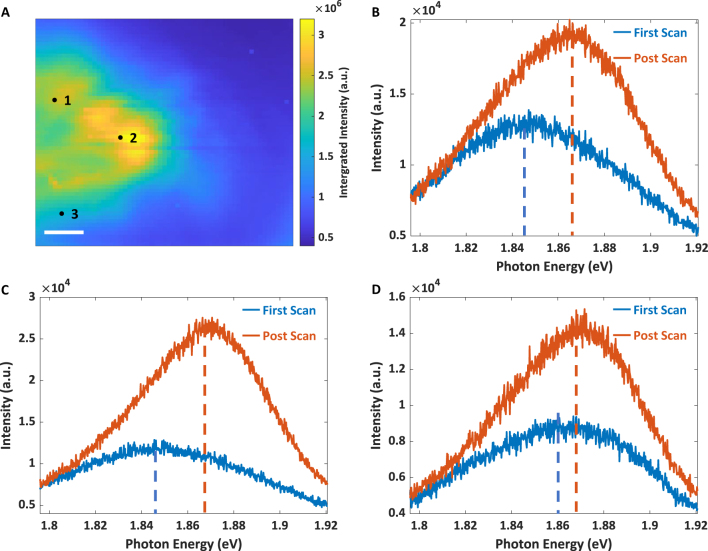
TEPL image of mechanically exfoliated monolayer MoS_2 _and spectra changes after multiple scans under bias voltages. (A) TEPL image of the final measurement with 0 V. The scanning area are the same as that in Figure 3A–C. (B–D) PL spectra (2 × 2 pixels) at position 1, 2, and 3, respectively. The blue curves represent spectra acquired during the first measurement with 0 V. The red curves represent spectra acquired during the final measurement with 0 V. The final measurement is conducted in around 24 h after applying +10 V and −10 V voltages. The dashed lines indicate the centers of the peaks of each spectrum. (B) 1.845 eV and 1.866 eV. **C**, 1.845 eV and 1.866 eV. (D) 1.860 eV and 1.867 eV.

To further understand this phenomenon, we conducted TEPL on mechanically exfoliated MoS_2_ with an Ag-coated tip. We labelled three positions, which locate on different MoS_2_ monolayer flakes, throughout the entire scanned area, as shown in Figure 3. To interpret the changes in the MoS_2_ flasks under bias voltages, we conducted experiments in the following sequence: (1) we scanned the first AFM image (Figure 1D) using a Si tip; (2) we measured the TEPL image with 0 V bias voltage for the original PL spectra (Figure 3A); (3) then flakes were measured with a bias voltage of +10 V applied to the tip, as shown in Figure 3B; (4) next, we acquired the TEPL image, shown in Figure 3C, under a bias voltage of −10 V; and (5) following the two scans with bias voltages, another TEPL was performed in 24 h under 0 V bias voltage to compare with the original PL spectra (Figure 4A).

Figure 3 shows the TEPL spectra of three points selected on MoS_2_ flakes under different voltages. Figure 3D–F demonstrate that when +10 V bias voltage was applied to the tip, the TEPL spectra (yellow curves) at all three positions, labelled in Figures 3A–C, displayed minor changes under a 2 s exposure time. In contrast, when the bias voltage was switched to −10 V, which reverses the direction of the electric field between the tip and the substrate, the TEPL intensities increased at all three positions, and the peak centers (red curves) were significantly blue shifted at positions 1 and 2. It appears that the changes in PL spectra are not solely due to the enhanced near field, tip temperature, or physisorption and chemisorption of molecules in the air [1, 28], [29], [30], [31]. Instead, the mechanism may be associated with the hot electrons generated by the LSPR process from the metal-coated tip during measurement, while bias voltages are responsible for removing the potential barrier between the tip and the MoS_2_ flake. As is shown in Figure 3F, the peak center of the TEPL spectrum at position 3 is not affected much by bias voltages during scans, indicating nonuniform contact potential barriers over MoS_2_, which is in consistent with our prior observations on CVD-grown MoS_2_ (see Discussion).

We investigated the mechanism behind the blue-shifted peak by measuring TEPL spectra of the same area without bias voltage, approximately 24 h after the first TEPL measurement (Figure 4). In comparison to the image obtained in the first TEPL scan, the post measurement is dramatically different under the same experimental conditions. Most areas show an increase in PL intensity after the two TEPL scans with nonzero bias voltages. The blue lines and red lines are TEPL spectra acquired before and after applying bias voltages to the tip, respectively. The intensities of PL spectra at positions 1 and 2 in Figure 4B and C are increased, and the peak centers of spectra are markedly blue shifted. Position 3 also exhibits an increase in the PL intensity, but the peak center shows a smaller blue shift. As observed during the postscan, all the three positions present similar PL peak positions, which are similar to the results observed on the CVD-grown MoS_2_ flakes. We consider MoS_2_ samples that have a uniform and unchanged PL reach a saturated state. More details are shown in the Supplementary Material 1. According to Figure 4, the changes in the PL of the MoS_2_ monolayer flakes, which include an increase in PL intensities and a blueshift in peak centers, endure for at least 24 h. Further experiment shown in the Supplementary Material 2 demonstrate that this saturated state can last over 6 months. It is anticipated that the peak center would exhibit a blue shift approaching to that observed for MoS_2_ exciton.

By comparing Figure 3D–F with Figure 4B–D, we observe that all the PL spectra in the final measurement under 0 V bias voltage at the three positions (1.862 eV, 1.866 eV, and 1.867 eV) are slightly blue shifted from the peak centers measured with the bias voltage of −10 V (1.854 eV, 1.859 eV, and 1.863 eV). The Ag tip can be stimulated to inject hot electrons into the MoS_2_ monolayer flakes under resonant excitation without bias voltages, and the bias voltage can speed up this process.

## 3 Discussion

As a result of the plasmon-induced hot electron injection, the PL signals of the edge and defect areas are enhanced and are blue shifted toward the PL signal of pure MoS_2_. TEPL measurements under resonant excitation lead to blue shifts in PL spectra. A negative voltage applied to the tip during the TEPL measurement can enhance this phenomenon, since the bias voltage overcomes the Schottky barrier between the tip and the MoS_2_ monolayer flake. On the contrary, applying a positive voltage to the tip during the measurement results in less apparent enhancement and blue-shift effects since the reversal potential blocks the hot electron injection. We think PL enhancements are caused by increasing electron densities in the conduction band as a result of the bias voltage. Similar reasons can be attributed to the increasing electron energy in the conduction band, which is responsible for the blue shift in PL.

Once the PL signal has reached saturation and is uniformly distributed over the MoS_2_ flake, more electrons will not cause further changes to the spectrum. The saturation is related to the maximum PL wavelength of exciton, which is determined by the limit of the MoS_2_ conduction band. Due to the imperfect crystalline structure, defects and MoS_2_ flake edges have more unoccupied states, making them more susceptible to hot charges. It is, therefore, evident that PL enhancement and blue shifts are more prominent in the area of the defect and edge.

All of these changes require a certain amount of exposure time in order to reach saturation. As long as the PL spectrum reaches saturation, it can remain unchanged for over 6 months, as shown in Figures 4 and S2. While the electrons are bound within the MoS_2_, the Si/SiO_2_ substrate also assists in maintaining them. Consequently, our technique fixes the optical response of defects and edges of MoS_2_ monolayer flakes and maintains the changes for a relatively long period of time.

In conclusion, we use TEPL to study the optical response of MoS_2_ monolayer flakes and provide an approach to fixing heterogeneous PL signals caused by defects and edges. Plasmon-induced hot electron injection leads to the enhancement and blue shift of PL in defect and edge areas. We have demonstrated that by adding a negative bias voltage to the tip, the PL signal will be saturated and become close to that of monolayer MoS_2_. Finally, we have evaluated the persistence of the fixed optical response, which lasts for a long time. The present work demonstrates a simple method for tuning the optical response of MoS_2_ monolayer flakes and paves the way to fixing the effects of defects and edges, which will benefit future studies of TMDCs with unavoidable defects.

## Supplementary Material

Supplementary Material Details
