# Supplementary material for: Tip-enhanced photoluminescence of monolayer MoS2 increased and spectrally shifted by injection of electrons
Source: Nanophotonics. 2023 Apr 6;12(14):2937–43. doi: 10.1515/nanoph-2023-0025 (PMC11501787; doi:10.1515/nanoph-2023-0025)
Supplement: Supplementary file 1 — Supplementary Material Details [file j_nanoph-2023-0025_suppl_001.pdf]

Tip-enhanced photoluminescence of monolayer MoS_2 increased and spectrally shifted by injection of electrons

Jizhou Wang[†1], Zehua Han[†1], Zhe He*[1,2], Kai Wang[1], Xiaohui Liu[3], Alexei V. Sokolov*[1,4]

[1]Institute for Quantum Science and Engineering, Texas A&M University, College Station, TX 77843, USA

[2]Department of Medical Engineering, California Institute of Technology, Pasadena, CA 91125, USA

[3]Department of Physics University of Texas at Austin, Austin, TX 78712, USA

[4]Baylor Research Innovative Center, Baylor University, Waco, TX 76798, USA

† These authors contributed equally.

* Correspondence should be addressed to Z. H. (hezhe@caltech.edu) and A. V. S. (sokol@tamu.edu).

S1 Voltage Dependence and the Saturated State

To demonstrate the evolution of the PL under bias voltage during the near-field scans, we scanned the same area of monolayer MoS_2 flakes 13 times under the same bias voltage (-2 V) with 1 s exposure time. The changes of tip-enhanced photoluminescence (TEPL) spectra are shown in figure S1. It is clearly shown that the peak position of the PL spectrum gradually blue-shifted until saturation when we scanned the MoS_2 sample.

To further study the changes caused by different bias voltages, we scanned 8 times (14-20 scan times in figure S1 B) more with different voltages (-4 V to -10 V) after reaching saturation. The 14th and 15th scan times were under -4 V; the 16th and 17th scan times were under -6 V; the 18th and 19th scan times were under -8 V; the 20th scan times were under -10 V. But no significant changes were observed.

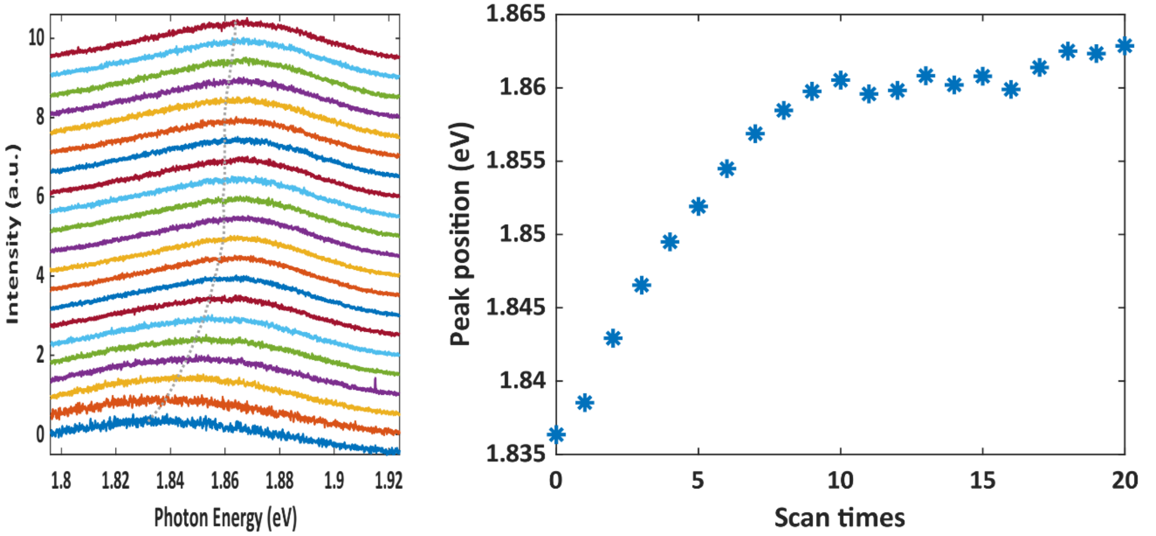

Figure S1. 21 TEPL spectra measured at the same spot on a mechanically exfoliated MoS_2 flake under different bias voltages. (A) 21 experimental TEPL spectra. These spectra are plotted from the bottom to the top followed by the scanning sequence. The first scan was under 0 V bias voltage for reference with 1 s acquisition time. All others were acquired under -2 V bias voltage with 1 s exposure time. (B) Peak positions of PL spectra shown in figure (A). X-axis is the scan times under non-zero bias voltages. The 0 time is the scan under 0 V for reference. The 1st to 13th scan times were under -2 V; the 14th and 15th scan times were under -4 V; the 16th and 17th scan times were under -6 V; the 18th and 19th scan times were under -8 V; the 20 scan times were under -10 V.

S2 TEPL Spectra after 6.5 Months

To determine the duration of the changed PL, we conducted measurements on the same area of the mechanical exfoliated sample under 0 V bias voltage approximately 6.5 months after the original experiment. Figure S2(B) shows the results of this measurement. The blue line, labeled '6.5 months,' represents the TEPL spectrum measured near the 'point 1' marked in figures 3 and 4, with a 1-second acquisition time. The red line, labeled 'Saturation,' is the PL spectrum measured at the 'New Point' in figure S2 when it was saturated (the 13th scan time in figure S1). It's important to note that both of these peaks were measured on the same MoS_2 flake, as shown in figure S2(A). Point 1 was almost saturated during the first time we did the experiment, and its TEPL spectrum shows that even after 6.5 months, it remained at the saturated status.

The new experimental data clarifies that the blue shift observed in the TEPL spectra indeed remained over a period of at least 6.5 months.

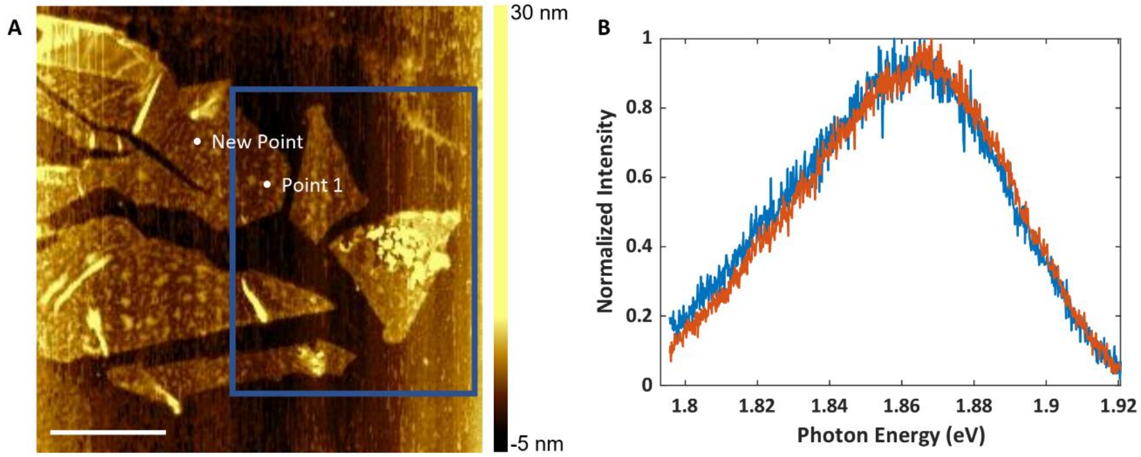

Figure S2 AFM image and TEPL spectra of mechanically exfoliated MoS_2 flakes after 6.5 months. (A) AFM image of mechanically exfoliated MoS_2 flakes. The blue rectangle is the scanned area shown in the main paper. ‘Point 1’ is near point 1 in the main paper (figure 3 and 4). The ‘new point' is where the voltage-dependence experiment (figure S1) is conducted. Its spectra are shown in figure S1. The scale bar is 1 µm. (B) TEPL spectra with 1 s acquisition time. The blue line is the TEPL spectrum measured around point 1 after 6.5 months since the original experiment reported in the main paper. The red line is the spectrum of the 13th scan shown in figure S1, which was saturated during scans. These two selected points are on the same MoS_2 monolayer flake.
